# Spontaneous, Loculated, and Massive Hemothorax: An Uncommon Complication of Warfarin Therapy

**DOI:** 10.7759/cureus.14923

**Published:** 2021-05-09

**Authors:** Ammar Al-Obaidi, Nicholas Tuck, Daly Al-Hadeethi, Alaeldin Mohammed, Quoc Truong

**Affiliations:** 1 Internal Medicine, University of Kansas School of Medicine, Wichita, USA; 2 Internal Medicine/Hospital Medicine, Wesley Medical Center, Wichita, USA; 3 Internal Medicine, Robert J. Dole Veterans Affairs Medical Center, Wichita, USA; 4 Cancer Center of Kansas, University of Kansas School of Medicine, Wichita, USA

**Keywords:** warfarin reversal, supratherapeutic inr, hemothorax, spontaneous hemothorax, warfarin therapy

## Abstract

Warfarin, a commonly used oral anticoagulant, is associated with several adverse drug reactions, principally bleeding. Of all hemorrhagic complications from warfarin therapy, thoracic hemorrhage accounts for only 3% and is usually related to trauma. Cases of spontaneous hemothorax secondary to anticoagulation therapy are rarely reported in the literature.

## Introduction

A hemothorax is defined as a pleural effusion with a hematocrit of at least 50% that of the peripheral blood. The majority of hemothorax cases are either chest trauma-related (open or closed) or iatrogenic. A spontaneous hemothorax is much less common [1].

Warfarin, a commonly used oral anticoagulant, is associated with several adverse drug reactions, principally bleeding. Of all hemorrhagic complications from warfarin therapy, thoracic hemorrhage accounts for only 3% and is usually related to trauma. Important risk factors for major hemorrhage due to warfarin therapy include a history of gastrointestinal bleeding, concurrent use of antiplatelet or nonsteroidal anti-inflammatory drugs, genetic differences in warfarin metabolism, international normalized ratio (INR) variability, comorbidities, and duration of oral anticoagulant therapy [2,3].

Spontaneous hemothorax from anticoagulant therapy is rarely reported in the literature, and the following highlights a case from warfarin use.

## Case presentation

A 64-year-old male presented in the ambulatory setting for routine follow-up and monitoring labs for his chronic medical conditions. At that time, his INR was found to be supratherapeutic at 13.31. His primary care provider recommended immediate hospital evaluation. On initial presentation to the emergency department, the patient was noted to be primarily asymptomatic and breathing comfortably. However, his vital signs revealed tachycardia and hypotension with a recorded blood pressure of 91/46 mmHg. His past medical history included atrial fibrillation with warfarin used as anticoagulation, cerebrovascular accident, hypertension, diabetes mellitus, seizures, and end-stage renal disease on hemodialysis.

On admission, warfarin was held and 5 mg of vitamin K was given. An initial hemoglobin level of 6 g/dL was noted, which required one unit of packed red blood cells. Vital measurements, physical examination, and his remaining biochemical profiles were unremarkable. No signs or symptoms of any obvious acute pathological bleeding was noted. The patient denied any history of trauma.

**Figure 1:**
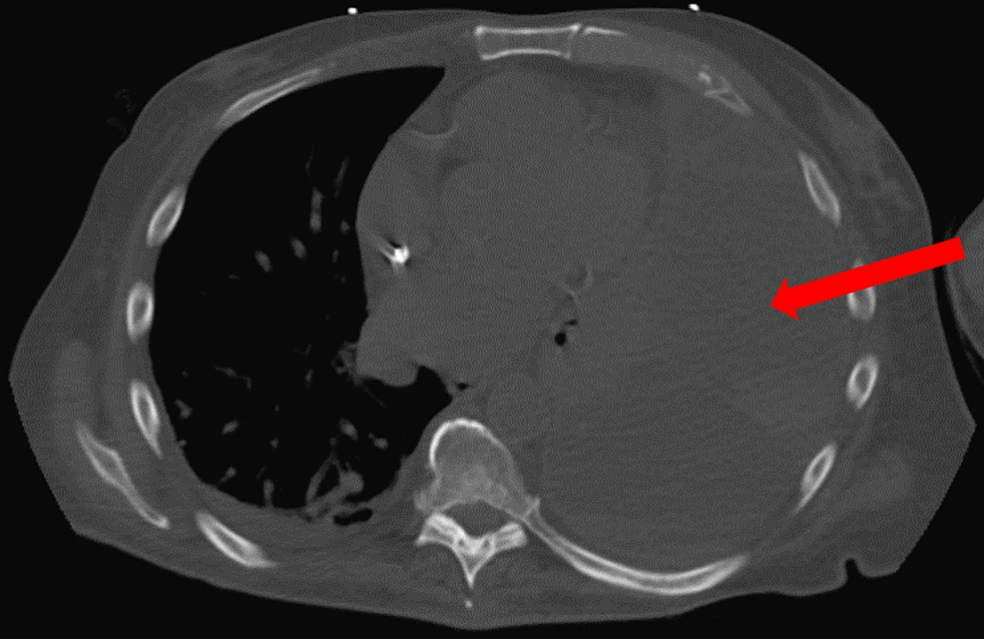
CT scan of the chest showing massive left-sided pleural effusion (red arrow). CT: computed tomography

Subsequent computed tomography (CT) scan revealed massive, left-sided pleural effusion (Figure 1). A previous chest X-ray obtained two months prior was unremarkable.

**Figure 2:**
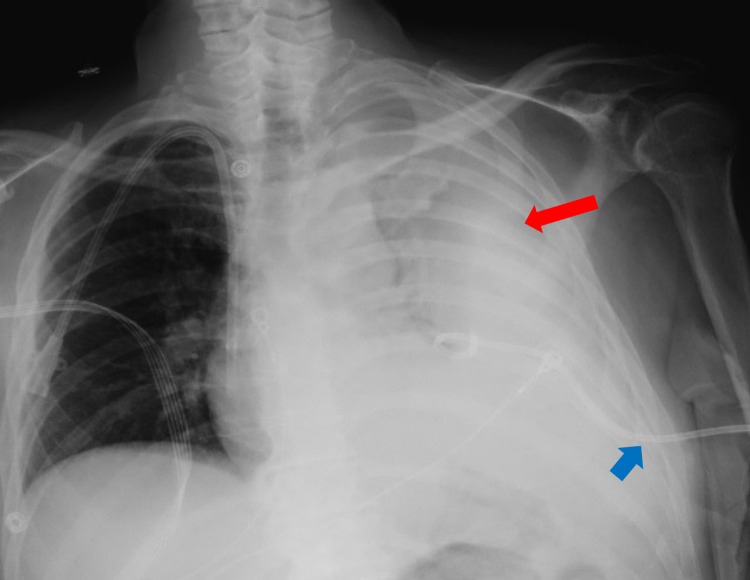
Chest X-ray showing complete opacification of the left hemithorax (red arrow) after chest tube insertion (blue arrow).

**Figure 3:**
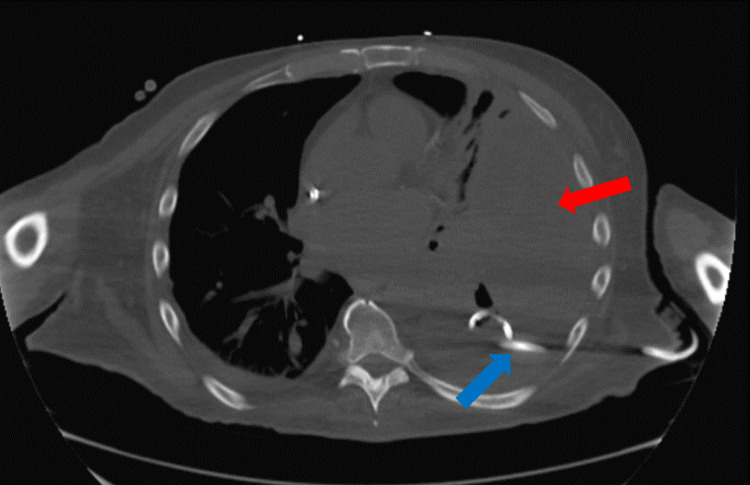
CT scan of the chest showing left-sided, large, and loculated effusion (red arrow) with chest tube in place (blue arrow). CT: computed tomography

Four-factor prothrombin complex concentrate was given to quickly reverse the INR. A chest tube was inserted and drained 650 mL of dark sanguineous fluid. Subsequent chest X-ray showed a persistent opacity over the left lung field (Figure 2); a repeat CT scan revealed a large loculated hemothorax (Figure 3).

Tissue plasminogen activator was administered through the chest tube, which resulted in minimal output. Thus, more invasive treatments were required. Video-assisted thoracoscopic surgery was unsuccessful and a full thoracotomy was performed, which resulted in left lower lobe wedge resection with decortication due to an air leak and a thick rind.

Laboratory studies done on the pleural fluid were unremarkable for infectious etiologies, including gram stain and cultures for bacteria, fungi, and acid-fast organisms. Cytology was also negative for malignant cells. Furthermore, the tissue biopsy showed no features of granulomas, dysplasia, neoplastic lesions, or significant inflammation.

## Discussion

Warfarin, a commonly used oral anticoagulant, is associated with several adverse drug reactions, principally bleeding. Of all the hemorrhagic complications from warfarin therapy, thoracic hemorrhage accounts for only 3% and is usually related to trauma. Massive hemothorax can present spontaneously in patients on oral anticoagulant therapy, especially in those with supratherapeutic INR. Pulmonary diseases including pleural pathologies are considered significant risk factors for developing a nontraumatic hemothorax in the setting of anticoagulation [4,5]. However, our patient had no previous history of pulmonary disease including malignancies of the lung itself or pleura, as well as no acute or chronic pulmonary emboli or aortic dissection. Workup for hematologic conditions was also unremarkable.

Hemothorax is a major indication for tube thoracostomy, particularly in cases with a mediastinal shift. However, significantly accelerated drainage can cause significant hypotension and resulting shock, thus caution is needed. Careful evaluation and monitoring of INR levels are required, especially if levels are supratherapeutic, as was seen in our patient [4]. Cessation of anticoagulant treatment must be the first step in treating anticoagulation-associated hemothorax followed by pharmacologic interventions to correct the coagulopathy before considering tube thoracostomy. Surgical intervention is dependent on individual patient circumstances.

## Conclusions

A massive hemothorax developing spontaneously in a patient whose only risk factor is oral anticoagulation therapy is an extremely rare finding. Elevated INR levels further increase the risk, and thus proper titration and adherence to medications are required. Treatment in these cases necessitates focused correction or reversal of coagulopathy caused by medications as well as tube thoracostomy for drainage. Further invasive procedures and/or operations require full utilization of the healthcare team.
